# Supplementary material for: Multi‐dataset identification of innovative feature genes and molecular mechanisms in keratoconus
Source: J Cell Mol Med. 2024 Sep 19;28(18):e70079. doi: 10.1111/jcmm.70079 (PMC11412914; doi:10.1111/jcmm.70079)
Supplement: Supplementary file 2 — Material S2. [file JCMM-28-e70079-s002.zip › 7.GSEA analysis/3.DBNDD1/KEGG_INOSITOL_PHOSPHATE_METABOLISM.html]

Details for gene set KEGG\_INOSITOL\_PHOSPHATE\_METABOLISM[GSEA]

|  || Dataset | DBNDD1.group.cls#h\_versus\_l |
| Phenotype | group.cls#h\_versus\_l |
| Upregulated in class | l |
| GeneSet | KEGG\_INOSITOL\_PHOSPHATE\_METABOLISM |
| Enrichment Score (ES) | -0.41730797 |
| Normalized Enrichment Score (NES) | -1.6169589 |
| Nominal p-value | 0.034548946 |
| FDR q-value | 1.0 |
| FWER p-Value | 0.553 |
Table: GSEA Results Summary

  

Fig 1: Enrichment plot: KEGG\_INOSITOL\_PHOSPHATE\_METABOLISM      
 Profile of the Running ES Score & Positions of GeneSet Members on the Rank Ordered List

  

| SYMBOL | TITLE | RANK IN GENE LIST | RANK METRIC SCORE | RUNNING ES | CORE ENRICHMENT || 1 | INPP4B | na | 5323 | 0.169 | -0.1186 | No |
| 2 | SYNJ1 | na | 6069 | 0.149 | -0.1094 | No |
| 3 | INPP5J | na | 6975 | 0.125 | -0.1098 | No |
| 4 | INPP1 | na | 8861 | 0.088 | -0.1460 | No |
| 5 | PLCB2 | na | 9009 | 0.086 | -0.1326 | No |
| 6 | PIP4K2B | na | 9747 | 0.075 | -0.1384 | No |
| 7 | PLCD3 | na | 10754 | 0.061 | -0.1548 | No |
| 8 | PTEN | na | 10959 | 0.059 | -0.1486 | No |
| 9 | MINPP1 | na | 11345 | 0.054 | -0.1486 | No |
| 10 | IPPK | na | 11697 | 0.050 | -0.1483 | No |
| 11 | PLCG1 | na | 11955 | 0.048 | -0.1460 | No |
| 12 | ALDH6A1 | na | 14027 | 0.030 | -0.1996 | No |
| 13 | PIKFYVE | na | 14064 | 0.030 | -0.1945 | No |
| 14 | INPP5K | na | 15249 | 0.021 | -0.2243 | No |
| 15 | PIK3C2B | na | 15826 | 0.017 | -0.2374 | No |
| 16 | PIP5K1C | na | 16052 | 0.015 | -0.2407 | No |
| 17 | PIK3CA | na | 17073 | 0.009 | -0.2683 | No |
| 18 | CDIPT | na | 17214 | 0.008 | -0.2707 | No |
| 19 | PLCD1 | na | 17682 | 0.005 | -0.2831 | No |
| 20 | PIK3CB | na | 18338 | 0.000 | -0.3019 | No |
| 21 | IMPA1 | na | 19145 | -0.006 | -0.3240 | No |
| 22 | INPP5A | na | 19288 | -0.007 | -0.3267 | No |
| 23 | PLCG2 | na | 19340 | -0.007 | -0.3267 | No |
| 24 | PLCB3 | na | 19605 | -0.010 | -0.3323 | No |
| 25 | IPMK | na | 19606 | -0.010 | -0.3304 | No |
| 26 | PLCB4 | na | 19707 | -0.010 | -0.3311 | No |
| 27 | TPI1 | na | 19904 | -0.012 | -0.3343 | No |
| 28 | PIK3C2A | na | 20995 | -0.022 | -0.3611 | No |
| 29 | ISYNA1 | na | 21052 | -0.023 | -0.3581 | No |
| 30 | PIK3C3 | na | 23106 | -0.044 | -0.4082 | Yes |
| 31 | PLCZ1 | na | 23423 | -0.048 | -0.4073 | Yes |
| 32 | INPPL1 | na | 23481 | -0.049 | -0.3988 | Yes |
| 33 | PIP4K2C | na | 23588 | -0.051 | -0.3913 | Yes |
| 34 | MIOX | na | 23665 | -0.052 | -0.3828 | Yes |
| 35 | IMPA2 | na | 24053 | -0.057 | -0.3822 | Yes |
| 36 | ITPK1 | na | 24085 | -0.057 | -0.3713 | Yes |
| 37 | PI4KB | na | 24192 | -0.059 | -0.3622 | Yes |
| 38 | PIK3C2G | na | 24699 | -0.066 | -0.3631 | Yes |
| 39 | PIK3CD | na | 24971 | -0.070 | -0.3565 | Yes |
| 40 | PIP4K2A | na | 25154 | -0.073 | -0.3467 | Yes |
| 41 | INPP5E | na | 25277 | -0.075 | -0.3347 | Yes |
| 42 | INPP4A | na | 25440 | -0.077 | -0.3234 | Yes |
| 43 | ITPKA | na | 25684 | -0.082 | -0.3136 | Yes |
| 44 | OCRL | na | 25862 | -0.085 | -0.3012 | Yes |
| 45 | PLCD4 | na | 26156 | -0.091 | -0.2909 | Yes |
| 46 | PIK3CG | na | 27455 | -0.118 | -0.3040 | Yes |
| 47 | ITPKB | na | 28941 | -0.155 | -0.3148 | Yes |
| 48 | SYNJ2 | na | 29282 | -0.165 | -0.2905 | Yes |
| 49 | PI4KA | na | 30046 | -0.190 | -0.2734 | Yes |
| 50 | INPP5B | na | 30273 | -0.197 | -0.2394 | Yes |
| 51 | PLCB1 | na | 32023 | -0.265 | -0.2352 | Yes |
| 52 | PIP5K1A | na | 32090 | -0.268 | -0.1818 | Yes |
| 53 | PLCE1 | na | 34128 | -0.447 | -0.1483 | Yes |
| 54 | PIP5K1B | na | 34702 | -0.803 | 0.0007 | Yes |
Table: GSEA details [plain text format]

  

Fig 2: KEGG\_INOSITOL\_PHOSPHATE\_METABOLISM      
 Blue-Pink O' Gram in the Space of the Analyzed GeneSet

  

Fig 3: KEGG\_INOSITOL\_PHOSPHATE\_METABOLISM: Random ES distribution      
 Gene set null distribution of ES for **KEGG\_INOSITOL\_PHOSPHATE\_METABOLISM**
